# Clinical impact of short limited lumbar fusion for adult spinal deformity with postural and radiological abnormalities

**DOI:** 10.1038/s41598-022-23933-z

**Published:** 2022-11-14

**Authors:** Hideaki Nakajima, Hideaki Matsuo, Hiroaki Naruse, Shuji Watanabe, Kazuya Honjoh, Kazuki Shoji, Arisa Kubota, Akihiko Matsumine

**Affiliations:** 1 Department of Orthopaedics and Rehabilitation Medicine, University of Fukui School of Medical Sciences, 23-3 Matsuoka Shimoaizuki, Eiheiji-cho, Yoshida-gun, Fukui, 910-1193 Japan; 2 Division of Physical Therapy and Rehabilitation Medicine, University of Fukui Hospital, 23-3 Matsuoka Shimoaizuki, Eiheiji-cho, Yoshida-gun, Fukui, 910-1193 Japan

**Keywords:** Outcomes research, Risk factors, Orthopaedics, Neurosurgery

## Abstract

Extensive surgical spinopelvic fusion for patients with adult spinal deformity (ASD) to achieve optimal radiological parameters should be avoided. The aim of this study was to review clinical and imaging findings in patients with ASD with postural and radiological abnormalities who underwent a novel three-level limited lumbar fusion as two-stage surgery in an attempt to propose a better tolerated alternative to spinopelvic long fusion to the pelvis. The subjects were 26 patients with a minimum follow-up period of 2 years. Cobb angle, C7 sagittal vertical axis, and pelvic incidence (PI) minus lumbar lordosis (LL) were significantly improved after surgery and maintained at follow-up. Most radiological parameters were corrected with lateral interbody fusion (LIF) as the initial surgery, and few with posterior fusion. PI-LL remained high after limited lumbar fusion, but scores on patient-based questionnaires and sagittal and coronal tilt in gait analysis improved. In cases with postoperative progression of proximal junctional kyphosis (11.5%), residual L1–L2 local kyphosis after LIF was the most significant radiological feature. In some cases of ASD with postural abnormalities, short limited lumbar fusion gives sufficient postoperative clinical improvement with preservation of spinal mobility and activities of daily living. The range of fixation should be determined based on radiological parameters after LIF to avoid postoperative complications.

## Introduction

Aging of the population has been accompanied by an increase in adult spinal deformity (ASD) linked to de novo degenerative lumbar scoliosis (DLS). The prevalence of DLS (Cobb angle ≥ 10°) has been found to be 35.5% in elderly subjects (≥ 60 years)^1^. Another study found an incidence of DLS of 36.7% over a mean period of 12.0 years^2^. Not all DLS cases are symptomatic, but approximately 40% of patients with radiographic DLS in the general Japanese population have been found to have symptoms, such as chronic low back pain (LBP) and lumbar spinal stenosis, with decreased function and quality of life (QOL)^3^.

Spinal deformity and global malalignment are common causes of postural instability and disabilities, and optimal radiological thresholds to improve QOL have been established for surgical planning for ASD^4^. Long fusion surgery such as thoracolumbosacroiliac arthrodesis is required in most cases with severe lumbar kyphoscoliosis. However, this surgery is highly invasive for elderly patients and has a high rate of postoperative complications^5^. Spinopelvic fixation may also limit activities of daily living (ADLs), such as sitting on the floor, farm work and dressing, due to decreased spinal mobility^6,7^. Long fusion is effective for improvement of radiological parameters, but the most important aim of surgery for ASD is to improve the condition with few postoperative complications and limited loss of ADLs. Thus, long segment instrumented fusion surgery for achievement of optimal radiological parameters should be avoided when possible.

Short fusion surgery is a reasonable option for patients with balanced de novo DLS without substantial sagittal imbalance^8^. However, the clinical outcomes of short limited lumbar fusion for patients with ASD with postural abnormality due to the intractable low back pain have not been examined. We hypothesized that a novel motion-sparing short limited lumbar fusion would work as well as historical long thoracolumbar fusions for well selected ASD patients. Thus, clinical and imaging findings after this surgery (3-segment fusion) for ASD were reviewed in this study to identify indications for this treatment.

## Results

### Changes in radiological parameters

Differences between preoperative and 3-week postoperative radiological parameters are shown in Table 1. Cobb angle, C7 SVA, and PI-LL were significantly decreased after surgery. Decreased coronal decompensation and increased LL were also found, but the difference was not significant. Radiological global alignment improved, but PI-LL was still high (> 10) after surgery. Changes in radiological parameters in the two-stage operation are shown in Table 2. Most radiological parameters were corrected during the 1st operation (LIF), and changes in the 2nd operation (posterior fusion) were small. The 1st operation did result in significant changes for the Cobb angle, L2 wedge angle, LL, L1–L2 angle, and PI-LL. Correction loss was observed at follow-up, but the median value was small. The inter- and intraobserver reliabilities for imaging findings were both excellent (p > 0.75).

**Table 1:** Demographic data and differences between pre- and postoperative (after 2nd surgery) radiological parameters.

Item	Preoperative	Postoperative	P
Age at operation, years	71.00 [64.75, 77.00]		
Male/female, n (%)	8 (30.8)/18 (69.2)		
Follow-up period, years	2.73 [2.40, 3.70]		
**Radiological assessments (º)**			
Cobb angle	13.90 [5.35, 19.45]	6.70 [1.65, 8.75]	0.048*
L2 wedge angle	6.10 [3.15, 13.70]	4.90 [1.90, 6.95]	0.29
Coronal decompensation	16.20 [10.75, 34.30]	7.10 [3.95, 16.65]	0.089
C7 sagittal vertical axis (SVA)	75.00 [46.95, 87.10]	52.40 [31.45, 61.30]	0.024*
Lumbar lordosis (LL)	11.60 [1.70, 27.20]	25.70 [16.75, 31.10]	0.078
L4–S1 angle	15.10 [10.25, 23.35]	15.90 [13.40, 19.70]	0.604
L1–L2 angle	− 3.70 [− 8.90, 2.05]	1.00 [− 1.00, 3.25]	0.115
Sacral slope	20.60 [11.45, 22.55]	22.80 [19.20, 24.15]	0.171
Pelvic incidence (PI)	49.10 [42.90, 52.50]	45.00 [37.85, 54.75]	0.547
PI-LL	38.20 [24.20, 42.54]	22.70 [15.80, 25.40]	0.01*

Data are shown as median [interquartile range].

*Significant difference for before vs. after surgery.

**Table 2:** Changes in radiological parameters in two-stage surgery.

Item	Postop-1st − preop	Postop-2nd − postop-1st	P	Correction loss at follow-up
Δ Cobb angle	−7.00 [−9.50, −3.40]	−0.20 [−1.40, 0.70]	0.001*	2.20 [−0.30, 4.30]
Δ L2 wedge angle	−1.80 [−4.50, −0.75]	−0.50 [−1.15, 0.55]	0.029*	0.25 [−0.50, 1.85]
Δ Coronal decompensation	−4.70 [−13.25, −0.15]	−1.40 [−10.15, 0.00]	0.589	1.50 [−0.08, 3.10]
Δ C7 SVA	−11.00 [−36.15, −0.70]	−1.80 [−5.90, 0.00]	0.089	2.10 [−2.10, 13.90]
Δ Lumbar lordosis (LL)	4.90 [3.14, 16.55]	1.10 [−2.40, 2.05]	0.009*	1.90 [−0.70, 3.72]
Δ L4–S1 angle	1.50 [−2.55, 6.00]	0.00 [−3.05, 1.95]	0.309	2.05 [−1.40, 4.90]
Δ L1–L2 angle	4.50 [−0.25, 8.80]	0.30 [−1.70, 0.55]	0.022*	−1.00 [−3.65, 1.30]
Δ PI-LL	−10.28 [−23.55, −3.15]	−0.40 [−2.50, 1.95]	0.004*	0.45 [−2.65, 3.67]

Data are shown as median [interquartile range].

*SVA* sagittal vertical axis, *PI* pelvic incidence.

*Significant differences for postop-1st vs. preop and postop-2nd vs. postop-1st.

### Clinical outcomes and changes in gait parameters

**Figure 1:**
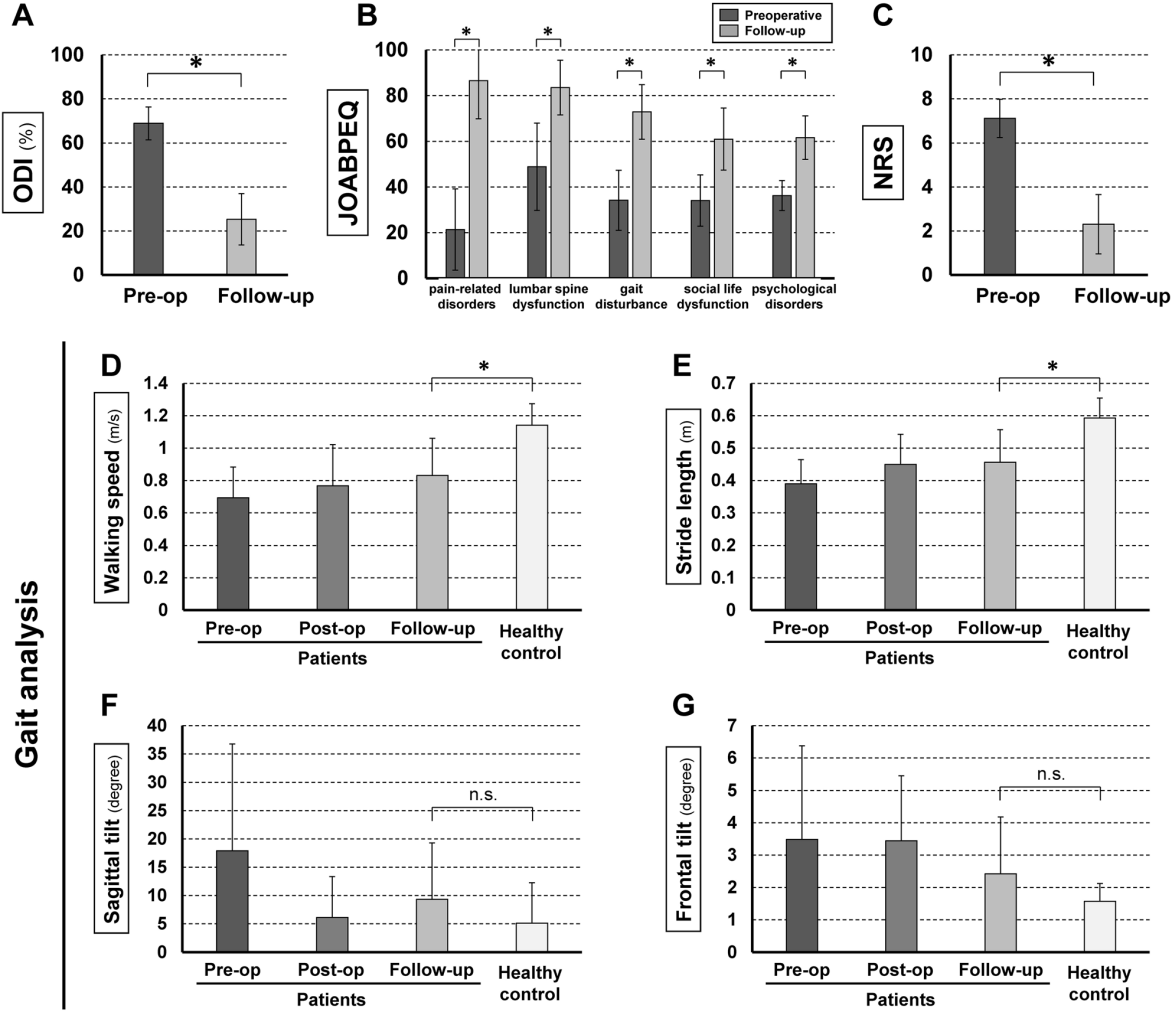
Clinical outcomes. (**A–C)** Patient-based questionnaires. Scores on the Oswestry Disability Index (ODI), Japanese Orthopedic Association Back Pain Evaluation questionnaire (JOABPEQ), and Numerical Rating Scale (NRS) were significantly improved at follow-up. (**D–G)** Gait analysis. Walking speed, stride length, sagittal tilt, and frontal tilt all improved, but none of these parameters reached the level found in healthy subjects. *p < 0.05. *n.s.* not significant.

Clinical outcomes based on the ODI, JOABPEQ (all subscales) and NRS (Fig. 1A–C) and gait parameters (Fig. 1D–G) all significantly improved at final follow-up, and there was no loss of ADLs for any patient after surgery. Mean walking speed, stride length, sagittal tilt, and frontal tilt in gait analysis all showed trends toward improvement, but did not reach the levels in healthy subjects in walking speed and stride length. At final follow-up, sagittal tilt showed a trend to being slightly increased compared with that after the 2nd operation.

### Postoperative complications and related factors

Postoperative complications occurred in three patients (11.5%), including progression of proximal junctional kyphosis (PJK) associated with loosening and migration of pedicle screws. Revision surgeries of conversion to long fusion from the thoracic spine were performed in two of these patients. The notable radiological features in cases with postoperative complications (Table 3) included residual L1–L2 local kyphosis after the 1st operation (LIF). All patients had L4–S1 kyphosis preoperatively, and 17 (65.4%) had L1–L2 local kyphosis, which disappeared after LIF in all but the three cases with complications. These three cases also had less correction of the Cobb angle and LL after LIF, compared to cases without complications.

**Table 3:** Relationships between postoperative complications and changes in radiological parameters.

Item	Stage	Without complication^a^	With complication		
			Case 1	Case 2	Case 3
Pelvic incidence	Preoperative	49.45 [47.12, 55.15]	49.9	37.9	35.8
Cobb angle (º)	Preoperative	7.30 [4.80, 14.68]	**32.2**	**23.6**	**16.6**
	After 1st operation	5.50 [1.77, 7.97]	**22**	**19.6**	**7.5**
	After 2nd operation	6.55 [1.20, 8.12]	**19.3**	**16.9**	**8.2**
Lumbar lordosis (º)	Preoperative	23.10 [10.22, 30.60]	**−6.6**	**−9.9**	**−5.5**
	After 1st operation	27.35 [19.72, 34.08]	**10**	**10.5**	**13.2**
	After 2nd operation	28.10 [21.27, 31.93]	11.1	18.8	18
PI-LL	Preoperative	30.80 [17.38, 38.73]	**43.3**	**47.8**	**51.3**
	After 1st operation	18.95 [14.80, 28.73]	23.0	24.4	22.9
	After 2nd operation	22.45 [12.98, 26.55]	19.8	23.2	20.9
L1–L2 angle (º)	Preoperative	0.30 [**−**5.45, 2.88]	**−**8.3	**−**6.8	**−**15.3
	After 1st operation	2.80 [1.23, 4.25]	**−8.5**	**−8.3**	**−4**
	After 2nd operation	1.55 [0.73, 4.17]	**−10**	**−7.1**	**−7.2**

Bold values indicate notable data.

^a^Data are shown as median [interquartile range].

### Representative cases

**Figure 2:**
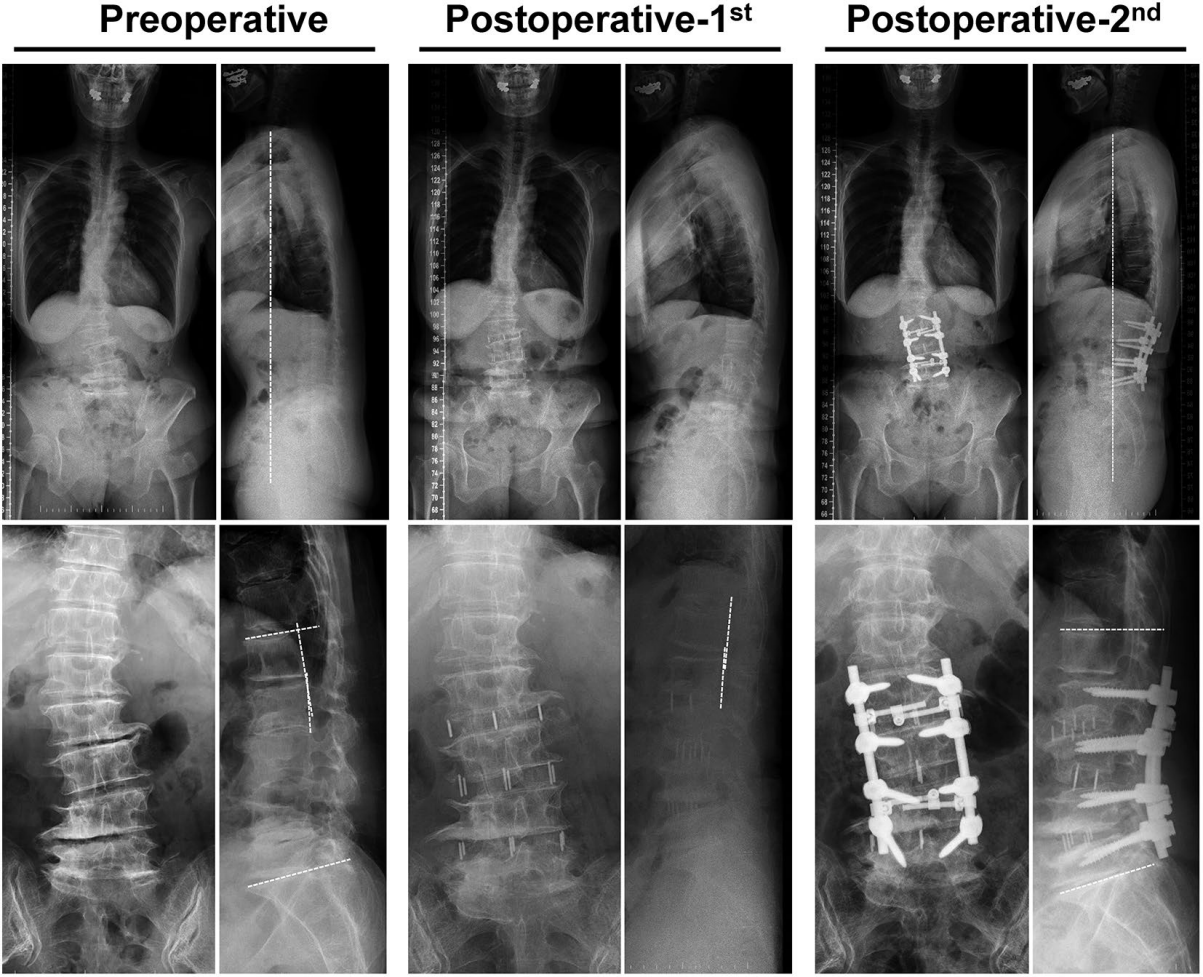
A 78-year-old female with severe low back pain and gait disturbance. Preoperative radiological parameters showed poor global alignment with C7 SVA 80.4 mm, LL −2.7°, L1–L2 angle −4.1°, and PI-LL 40.3°. The L1–L2 angle was 1.4° after the 1st operation. There were improvements to C7 SVA 25.9 mm, LL 19.6°, and PI-LL 21.9° at 2 years after surgery.

**Figure 3:**
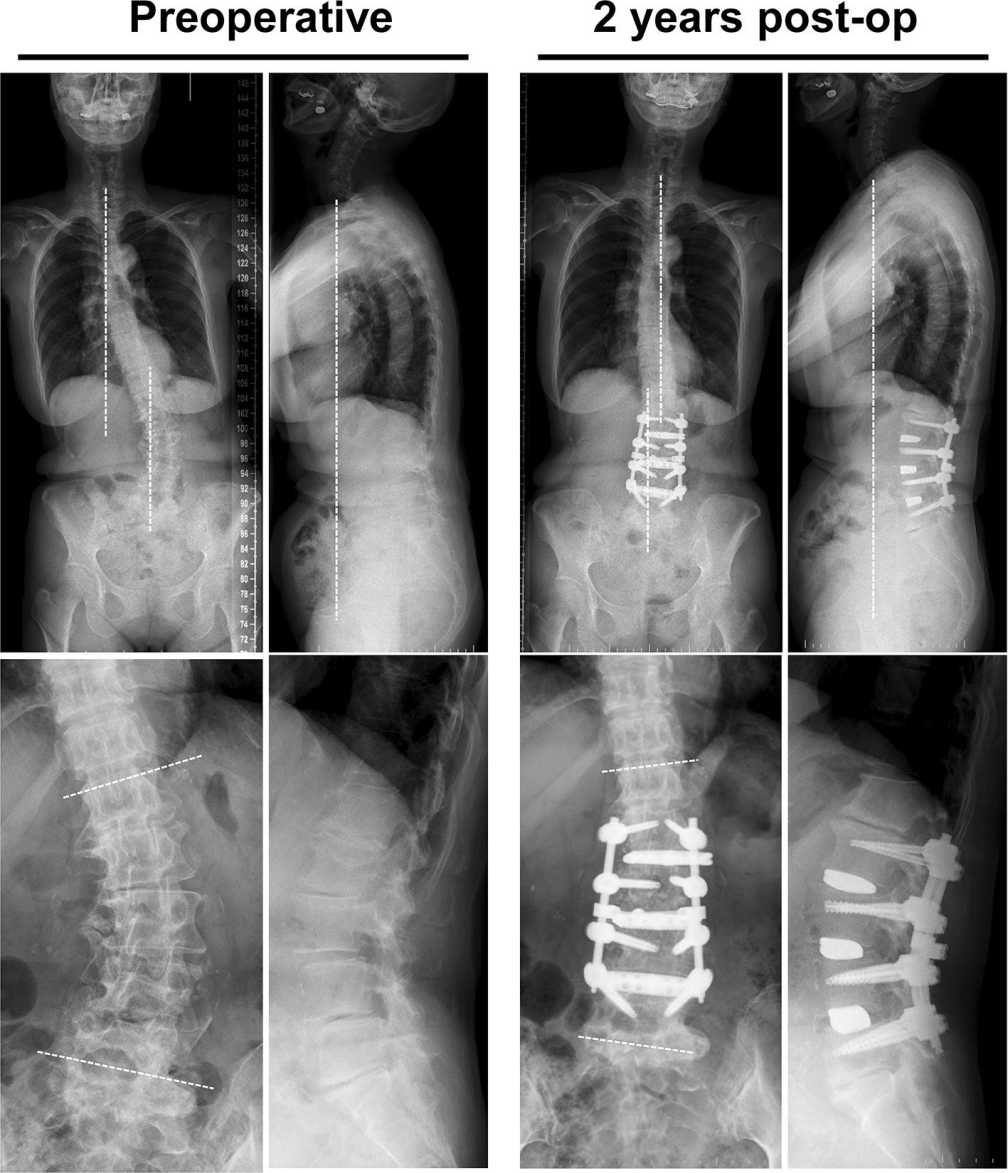
A 68-year-old female with gait disturbance mainly due to frontal trunk tilt. Preoperative radiological parameters showed poor global alignment with Cobb angle 30.6°, coronal decompensation 59.9 mm, C7 SVA 116 mm, and PI-LL 18.4°. These parameters improved to 1.4°, 6.1 mm, 62.5 mm, and 15.9°, respectively, at 2 years after surgery.

**Figure 4:**
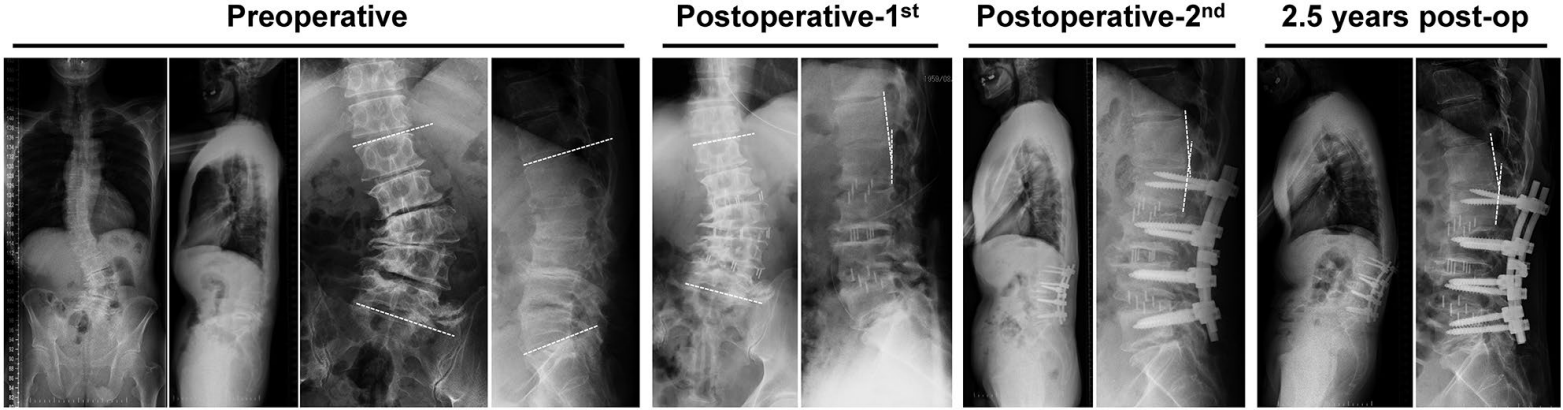
A 59-year-old male with low back pain and gait disturbance due to postural abnormalities. Preoperative radiological parameters showed poor global alignment with Cobb angle 32.2°, coronal decompensation 27.7 mm, LL 6.7°, L1–L2 angle −8.3°, and PI-LL 33.3°. Residual L1–L2 local kyphosis was apparent after the 1st and 2nd operations, and progressive proximal junctional kyphosis with severe low back pain appeared postoperatively.

**Figure 5:**
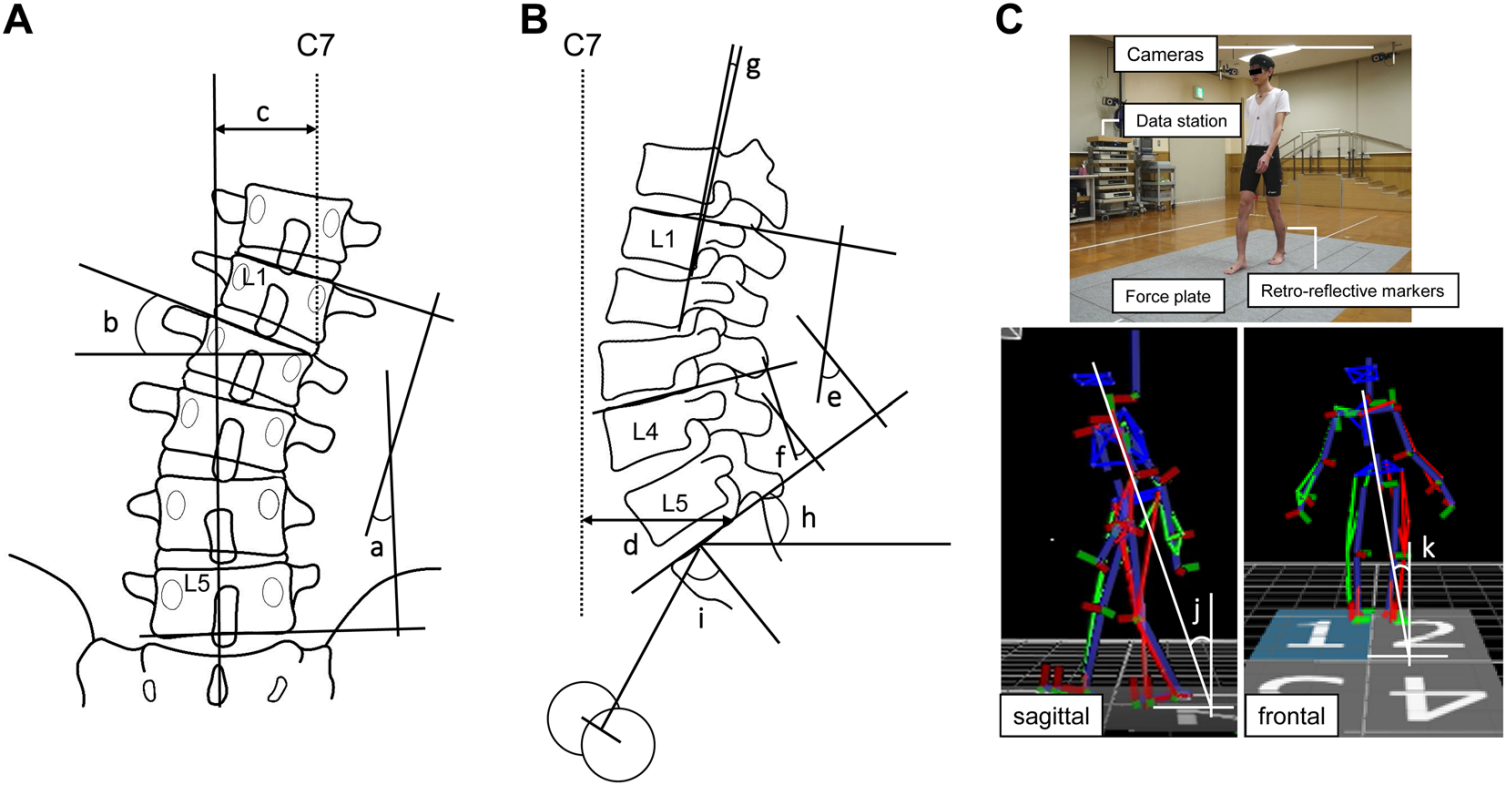
Radiological measurements and gait analysis. (**A**) Lumbar coronal plain radiographs: (a) Cobb angle (L1–L5), (b) L2 wedge angle, (c) coronal decompensation using the C7-central sacrum vertical line (CSVL). (**B**) Lumbar lateral plain radiographs: (d) C7-sagittal vertical axis (SVA), (e) lumbar lordosis (LL) (L1–S1), (f) L4–S1 angle, (g) L1–L2 local lordotic angle, (h) sacral slope, (i) pelvic incidence (PI). (**C)** Gait analysis using a 3D motion analysis system. Walking speed and step length were assessed as spatiotemporal parameters. Sagittal and coronal tilt (angle of the thorax segment relative to the global coordinate system) were assessed as kinematic parameters.

Pre- and postoperative radiological images from three representative cases are shown in Figs. 3, 4 and 5. The first patient (Fig. 2) was a 78-year-old female with LBP and gait disturbance due to postural abnormalities. Preoperative radiological parameters showed poor global alignment with C7 SVA 80.4 mm, LL −2.7°, L1–L2 angle −4.1°, and PI-LL 40.3°. The L1–L2 angle was 1.4° after the 1st operation, and the radiological parameters improved to C7 SVA 25.9 mm, LL 19.6°, and PI-LL 21.9° at 2 years postoperatively. In gait analysis, sagittal tilt significantly improved (43.3° → 17.5°), along with walking speed (0.57 → 0.68 m/s), stride length (0.38 → 0.46 m), and frontal tilt (3.6° → 1.3°) (see Supplementary Video S1 online). ODI (71.1% → 26.7%), JOABPEQ, and NRS (8 → 2) scores also improved.

The second patient (Fig. 3) was a 68-year-old female with gait disturbance due to postural abnormalities. Preoperative radiological parameters showed poor global alignment with Cobb angle 30.6°, coronal decompensation 59.9 mm, C7 SVA 116 mm, and PI-LL 18.4°. These radiological parameters improved to 1.4°, 6.1 mm, 62.5 mm, and 15.9°, respectively, at 2 years postoperatively. In gait analysis, frontal tilt (7.3° → 4.2°) markedly improved, along with walking speed (0.89 → 1.03 m/s), stride length (0.45 → 0.52 m), and sagittal tilt (28.7° → 16.6°) (see Supplementary Video S2 online). ODI (51.1% → 13.3%), JOABPEQ, and NRS (7 → 1) scores also improved.

The third patient (Fig. 4) was a 59-year-old male with LBP and gait disturbance due to postural abnormalities. Preoperative radiological parameters showed poor global alignment with Cobb angle 32.2°, coronal decompensation 27.7 mm, LL 6.7°, L1–L2 angle −8.3°, and PI-LL 33.3°. L1–L2 local kyphosis remained after the 1st and 2nd operations, and PJK with severe back pain was present at 2.5 years after surgery.

## Discussion

Sagittal spinopelvic alignment has been suggested to be important for pain and disabilities in patients with ASD, and the outcomes of surgery for ASD are dependent on sagittal imbalance^4,9^. However, corrective fusion surgery for ASD is fraught with problems such as a high incidence of complications and reoperation, particularly in elderly patients. A multicenter survey in Japan identified major complications in 14.5% of patients after corrective surgery for ASD, and patients aged ≥ 65 years had higher rates of deformity due to degeneration, as well as a higher incidence of complications^10^.

Some recent studies have suggested that spinopelvic long fusion does not always increase ADLs, but rather restricts ADLs that involve strenuous activities that require greater spinal mobility^6,7^. Thus, significantly reduced performance of activities such as weeding and farm work occurs postoperatively at rates of 42.1–87.5%. Other basic activities, such as trimming toe nails and picking up an item from the floor, also significantly deteriorated in 21–88% patients^11^. A multicenter retrospective study suggested that the correlation between sagittal spinal alignment and QOL is not as strong as previously reported in patients with ASD with de novo symptomatic DLS^12^. In the present study, the postoperative radiological parameters did not necessarily reach optimal values^4^. However, patient satisfaction on the ODI was significantly high and there was no loss of preoperative ADLs. There was also significant postoperative improvement in gait with mild residual trunk anteversion, as well as improvement of static radiological parameters. Gait analysis avoids underestimation of global sagittal alignment loss detected by static plain standing whole spine radiography^13^. These results indicate that there are ASD cases with postural abnormalities in which QOL can be improved with short limited lumbar fusion without loss of ADLs.

In spinopelvic long fusion surgery, LL can be obtained by pushing the bent rods into the center of the body using the cantilever technique, starting from the pelvis. In contrast, short limited lumbar fusion has a technical limitation in creating an optimal LL because correction is mainly performed by rod rotation. Thus, it has been suggested that short fusion may not improve QOL sufficiently for patients with PI > 47° because of persistent PI-LL mismatch^14^. On the other hand, another study found that a larger PI requires a less rigorous alignment than in a case with a smaller PI^15^. In the present study, 17 patients (65.4%) had PI > 47°, but the postoperative ODI was significantly improved in most cases. Furthermore, 2 of 3 patients with postoperative complications had preoperative PI < 47°, suggesting that there may be a large tolerance for global alignment in older patients with larger PI. Severity of preoperative spinal deformity is certainly a risk factor for postoperative complications. However, the results of this study suggest that the most important prognostic factor for complication might be the radiological parameters after the 1st operation, rather than preoperatively. A multicenter retrospective study suggested that sagittal spinopelvic alignment varied with age, and elderly patients who are more frail do not need to be held to as rigorous alignment objectives^16^. Thus, the surgical indication for ASD with de novo symptomatic DLS should not be overly influenced by abnormalities in radiological parameters, but should be determined based on preoperative possible ADLs and the needs of each patient.

Short fusion has been suggested to cause rapid progression of scoliosis^17^. Among all complications after spinal correction surgery, PJK is a common and critical pathology. In spinopelvic long-fusion surgery, extension of upper instrumented vertebra to T10 or above is recommended to minimize the risk of PJK^18^. A retrospective study including patients with DLS of mean age 68.3 years suggested that larger lumbar lordosis correction, higher age (≥ 75 years), and sacropelvic fusion were risk factors for postoperative PJK^19^. Overcorrecting LL, especially in elderly patients, can lead to mechanical complications such as PJK due to the effect against the natural shape of the spine^20^. From this point of view, correction of LL to reach optimal radiological parameters to prevent PJK should be avoided, especially in short limited lumbar fusion, as this may lead to L1–L2 local kyphosis.

In the present study, the median correction loss of the Cobb angle was 2.2°, and this increase of the Cobb angle was almost identical to that found in natural progression in ASD^21^. Patients with ASD with de novo DLS have variable degrees of lumbar degeneration and stiffness, and it is difficult to decide whether to perform spinopelvic long fusion or short limited lumbar fusion based on preoperative radiological parameters. From the results in the present study, a negative L1–L2 angle and a residual large Cobb angle and insufficient LL correction after the 1st operation (LIF) were predictors for postoperative complications. These findings suggest that determining the range of fixation after LIF in two-stage surgery might help to avoid long-segment fixation/fusion and reduce postoperative complications.

This study has certain limitations, including its retrospective, single center design, small number of patients, and a lack of comparison with patients with similar clinical backgrounds who underwent spinopelvic long fusion surgery. Thus, there is a need for prospective studies with longer follow-up periods to define the optimal surgical procedure. However, we suggest that, within these limitations, our results provide key insights into the appropriate surgical method for patients with ASD with postural abnormality.

In conclusion, among patients with ASD with postural abnormalities, there are cases indicated for short limited lumbar fusion that results in sufficient postoperative clinical improvement. These cases have (1) an absence of lumbar kyphosis (preserved L4-S1 lordosis) and thoracolumbar scoliosis, and (2) no residual local kyphosis at L1–L2 and sufficient correction of the Cobb angle and LL after LIF. Two-stage surgery is recommended to determine the range of fixation to avoid postoperative complications due to the difficulty in significant correction with limited-segment fusion via a posterior approach. Careful consideration of short or long fusion surgery is needed, but our results show that short lumbar fusion for ASD can provide sufficient improvement in QOL, including gait, while preserving spinal mobility and ADLs.

## Methods

### Study population and surgical indication

The subjects were 26 consecutive ASD patients (8 males and 18 females, median age of 71 years) presented with postural and radiological abnormalities who underwent 3-segment lumbar fusion (L2–L5) at our hospital between 2017 and 2019, and had a minimum follow-up period of 2 years (median 2.73 years) (Table 1). The surgical indications for 3-segment lumbar fusion for ASD during this period were determined (1) absence of lumbar kyphosis (preserved L4–S1 kyphosis), (2) scoliosis within the range of the lumbar spine, (3) absence of spinal canal stenosis at L5–S1, and (4) absence of symptoms due to L5–S1 intervertebral foraminal stenosis. Two-stage surgery was performed, with initial 3-segment lateral interbody fusion (LIF) (L2–L5), followed by posterior fixation with L2/3/4/5 facetectomy and bone grafting without decompression one week later. The cohort had no cervical myelopathy, trauma, or neurological diseases, which can occur with ASD.

### Radiological assessments

The following parameters were assessed to determine the spinopelvic alignment using plain standing whole spine and lumbopelvic radiography preoperatively, postoperatively (3 weeks after surgery), and at follow-up (≥ 2 years after surgery): Cobb angle (L1–L5), L2 wedge angle, coronal decompensation using the C7-central sacrum vertical line (CSVL), C7-sagittal vertical axis (SVA), lumbar lordosis (LL), L4–S1 angle, L1–L2 angle, sacral slope, pelvic incidence (PI), and PI-LL (Fig. 5A,B). All parameters were measured in triplicate by two observers, and the average was used for analysis. Preoperative radiological abnormality was defined as C7-SVA > 50 mm and PI-LL > 10°^4^. Preoperative foraminal stenosis and canal stenosis were assessed on sagittal, axial, and coronal lumbar CT and MRI.

### Gait analysis

Gait analysis was performed preoperatively, 3 weeks postoperatively, and at follow-up after ≥ 2 years using a 3D motion analysis system (VICON-MX, Oxford, UK) with strobe cameras and four force plates placed in the middle of a 10 m walkway (Fig. 5C). Thirty-five reflective markers were placed on anatomical landmarks according to the Plug-In-Gait marker set. Patients walked barefoot at their own pace without aids or a cane. Three patients who were unable to walk 10 m without aids preoperatively were excluded. Walking speed and step length were assessed as spatiotemporal parameters, and sagittal and coronal tilt (angle of the thorax segment relative to the global coordinate system) were assessed as kinematic parameters^22^. The mean tilt (3 trials) in one gait cycle was calculated for each patient. As a reference, gait analysis was performed in 6 healthy subjects of similar age (median 72 years).

### Patient-based questionnaires

To evaluate the effect of ASD on LBP and ADLs, three patient-based questionnaires were used: the Japanese version of the Oswestry Disability Index (ODI), excluding the question on sexual activity (0–100%)^23^, the Japanese Orthopedic Association Back Pain Evaluation questionnaire (JOABPEQ) (0–100 for 5 subscales)^24^, and the Numerical Rating Scale (NRS) (0–10). These questionnaires were administered preoperatively and at 2 years after surgery.

### Statistical analysis

Data are presented as median values [interquartile range] or mean ± SD. Comparisons between groups were performed by Mann–Whitney U-test or t-test, with P < 0.05 considered to be significant. Intraclass correlation coefficients (ICCs) were used to evaluate inter- and intraobserver reliabilities. EZR (Saitama Medical Center, Jichi Medical University, Saitama, Japan), a GUI for R (The R Foundation for Statistical Computing, Vienna, Austria), was used for all calculations.

### Ethical declarations

The study protocol was approved by the Human Ethics Review Committee of our University Medical Faculty (Approval Number 2014046) and strictly followed the Clinical Research Guidelines of the Ministry of Health, Labor, and Welfare of the Japanese Government.

### Consent for participate

Written informed consent was obtained from each patient and healthy subject.

## Supplementary Information

Supplementary Video 1.

Supplementary Video 2.

Supplementary Legends.

## Data Availability

Data generated and analyzed during this study are included in this published article. Data and materials are available from the corresponding author subject to reasonable request and subject to the ethical approvals in place and materials transfer agreements.
