# Supplementary material for: Clinical impact of short limited lumbar fusion for adult spinal deformity with postural and radiological abnormalities
Source: Sci Rep. 2022 Nov 14;12:19439. doi: 10.1038/s41598-022-23933-z (PMC9663600; doi:10.1038/s41598-022-23933-z)
Supplement: Supplementary file 3 — Supplementary Legends. [file 41598_2022_23933_MOESM3_ESM.docx]

**Supplementary Video Legends**

**Video S1.** Pre- and postoperative (2 years) gait analysis movie of the case shown in Figure 3. Walking speed and stride length improved from 0.57 to 0.68 m/s and 0.38 to 0.46 m; and sagittal and frontal tilt improved from 3.6° to 1.3° and 43.3° to 17.5°, respectively.

**Video S2.** Pre- and postoperative (2 years) gait analysis movie of the case shown in Figure 4. Walking speed and stride length improved from 0.89 to 1.03 m/s and 0.45 to 0.52 m; and sagittal and frontal tilt improved from 7.0° to 0.05° and 28.7° to 16.6°, respectively.
